# Retrospective analysis of multimorbidity, polypharmacy, and drug interactions on postoperative outcomes in oral squamous cell carcinoma patients

**DOI:** 10.1007/s00784-025-06588-8

**Published:** 2025-10-20

**Authors:** A. Schmitz, C. Reichardt, J. Gierth, M. Richter, F. Mrosk, M. Alfertshofer, L. Knoedler, C. Doll, F. Hausmann, M. Lee, B. Kleikamp, C. Rendenbach, M. Heiland, S. Koerdt

**Affiliations:** 1 Department of Oral and Maxillofacial Surgery, Charité – Universitätsmedizin Berlin, corporate member of Freie Universität Berlin and Humboldt-Universität zu Berlin, Augustenburger Platz 1, Berlin, 13353 Germany; 2 Department of Radiation Oncology, Charité – Universitätsmedizin Berlin, corporate member of Freie Universität Berlin and Humboldt-Universität zu Berlin, Augustenburger Platz 1, Berlin, 13353 Germany; 3 Department of Gynecology with Center for Oncological Surgery, European Competence Center for Ovarian Cancer Campus Virchow Klinikum and Charité Comprehensive Cancer Center (CCCC), Charité – Universitätsmedizin Berlin, corporate member of Freie Universität Berlin and Humboldt-Universität zu Berlin Department of Gynecology with Center for Oncological Surgery, European Competence Center for Ovarian Cancer Campus Virchow Klinikum and Charité Comprehensive Cancer Center (CCCC), Augustenburger Platz 1, Berlin, 13353 Germany; 4 Department of Anesthesiology and Intensive Care Medicine, Charité - Universitätsmedizin Berlin, Charité – Universitätsmedizin Berlin, corporate member of Freie Universität Berlin and Humboldt-Universität Zu Berlin, Hindenburgdamm 30, Berlin, 12203 Germany

**Keywords:** Oral squamous cell carcinomas, Multimorbidity, Polypharmacy, Drug interaction risk, Overall survival, Recurrence rates

## Abstract

**Objectives:**

This retrospective study investigates the impact of multimorbidity, polypharmacy, and drug interactions on postoperative outcomes and patient management in individuals diagnosed with oral squamous cell carcinoma (OSCC). The study aims to identify treatment-related risks within surgical care pathways and explore the role of pharmacological complexity in patient prognosis.

**Materials and methods:**

Clinical data from OSCC patients undergoing surgical treatment were retrospectively analyzed. Key parameters included the presence of multiple chronic conditions (multimorbidity), the number and types of prescribed medications (polypharmacy), and the use of potentially inappropriate medications (PIMs) according to the PRISCUS list. Primary outcomes included postoperative complications, wound healing, readmission, reoperation rates, and mortality.

**Results:**

Multimorbidity and polypharmacy were significantly associated with increased rates of postoperative complications, delayed wound healing, and higher rates of hospital readmission. PIMs were prescribed in 13.7% of patients and were linked to elevated mortality. Importantly, patients who did not receive PRISCUS-listed medications had a 55% lower risk of readmission and a 52.3% lower risk of reoperation, suggesting a potential benefit of medication optimization in perioperative care.

**Conclusions:**

The findings emphasize the relevance of multimorbidity and complex medication regimens in influencing surgical outcomes in OSCC. Systematic preoperative risk assessment and medication review are critical to reducing complications and improving recovery.

**Supplementary Information:**

The online version contains supplementary material available at 10.1007/s00784-025-06588-8.

## Highlights

- Prescribing potentially inappropriate medications (PIMs) in 13.7% of patients was associated with higher mortality and increased postoperative complications, while avoiding PRISCUS-listed drugs reduced rehospitalization risk by 55% and reoperation risk by 52.3%.
- Multimorbidity (≥ 2 chronic conditions) was present in 37.9% of the cohort.
- The study sheds light on the immunomodulatory activities of the HA/SA scaffold loaded with LUT-Zn nanocomposite within the immune microenvironment of the pressure ulcer wound.
- Hormonal and antineoplastic drugs significantly impacted overall survival.

## Introduction

Oral squamous cell carcinoma (OSCC) is the most prevalent malignancy of the head and neck region, accounting for approximately 90% of all oral cancers and representing a significant global health burden [1]. With global OSCC incidence exceeding 389,000 new cases annually, cases rose by 3.21% to 389,846, and deaths increased by 6.01% to 188,438 from 2020 to 2022; this trend is expected to worsen, with incidence projected to rise 65% by 2050 [2]. The disease predominantly affects individuals over 50 years of age, with rising incidence rates in older populations, partially due to cumulative exposure to established risk factors such as tobacco use, alcohol consumption and betel quid chewing [3]. Despite significant advancements in surgical techniques, adjuvant therapies, and early detection methods, the prognosis remains highly variable, with overall survival rates (OS) ranging from 40% to 60% depending on tumor stage, patient demographics and overall health status.

The management of OSCC is a particularly challenging aspect of cancer care in an aging population, where the prevalence of multimorbidity and polypharmacy is increasing. Multimorbidity, defined as the presence of two or more chronic health conditions, is highly prevalent among older adults and associated with adverse outcomes, including higher rates of hospitalizations, increased healthcare expenditures [4, 5], diminished patient adherence to treatment [6–8], and reduced OS [9, 10]. While the World Health Organization defines polypharmacy as the concurrent use of four or more medications [11], most academic studies adopt a threshold of five or more. In this Study, the threshold of ≥ 5 medications was selected in line with definitions commonly used in oncology and geriatric research, particularly in cancer patients, where polypharmacy is associated with adverse outcomes and reduced survival [12]. To ensure consistency with existing research, this study applied the academic definition of polypharmacy.

Polypharmacy, often a consequence of multimorbidity, introduces additional risks such as drug-drug interactions, adverse drug events, and diminished efficacy of cancer therapies [13].

The repercussions of polypharmacy and multimorbidity extend beyond systemic treatment complications, profoundly impacting geriatric pharmacotherapy and, in the context of OSCC patients, surgical outcomes with an elevated incidence of postoperative complications, encompassing infections, delayed wound-healing and protracted hospitalizations. Therefore, the PRISCUS list, introduced in Germany in 2010, was developed to identify potentially inappropriate medications (PIMs) in older adults, similar to the Beers criteria in the United States. Medications listed in the PRISCUS list are those considered potentially inappropriate for use in older adults due to increased risk of adverse drug events and interactions, especially in the context of polypharmacy and multimorbidity [14–16].

Given the predominantly advanced age of OSCC patients, this potential risk of drug interactions in represents a critical dimension of clinical complexity. Especially with these interactions influencing the pharmacokinetics and pharmacodynamics of anti-cancer therapies, their efficacy or exacerbating toxicities may be altered.

In addition to the direct impact of multimorbidity, polypharmacy and drug interaction risks on OSCC outcomes, these factors also have broader implications for healthcare systems. Multimorbid patients often require multidisciplinary care involving oncologists, primary care physicians, pharmacists, and other specialists, creating logistical and communication challenges. Moreover, the economic burden of managing multimorbidity and polypharmacy is substantial, with higher costs for medications, increased utilization of healthcare services, and longer hospitalizations. These factors underscore the need for integrated care models that address the unique needs of OSCC patients, ensuring coordinated, patient-centered approached to treatment.

Given these challenges, a thorough understanding of the interplay between multimorbidity, polypharmacy, and drug interactions is essential for improving patient management and treatment outcomes. This study seeks to analyze the associations between these factors and their impact on clinical course, survival, and recurrence in OSCC patients.

## Methods

### Study design

Between 2017 and 2022, 190 patients were enrolled in this retrospective cohort study conducted at the Department of Oral and Maxillofacial Surgery, Charité - Universitätsmedizin Berlin, Germany. All patients with primary OSCC who underwent curative surgical treatment during this period were included in the study.

### Ethics statement

Ethical approval was granted by the institutional ethics committee of Charité - Universitätsmedizin Berlin, Germany (reference number: EA2/077/20). As this was a retrospective study, obtaining patient informed consent was not required.

### Data collection

A systematically collected retrospective dataset included all patients diagnosed with primary OSCC (ICD-10: C00-C06) according to AJCC 8th Edition and treated curatively. It encompassed demographics (age, sex, BMI), comorbidities, survival metrics, treatment-parameters (postoperative complications, rehospitalization data), lifestyle factors (smoking, alcohol consumption), pharmacological profiles (polypharmacy, PRISCUS-listed medications, risk of drug-interactions) and treatment outcomes (reoperation, hospital readmission, discharge destinations, admission origin).

Exclusion criteria included incomplete resection, concurrent carcinoma, distant metastases at diagnosis, missing records of medication history, pre-existing conditions, or prior head and neck irradiation.

Patients were stratified by age (< 65 years vs. ≥65 years) to account for age-related clinical and pharmacological differences, particularly relevant in geriatric oncology [17].

Multimorbidity was defined as the presence of ≥ 2 systemic diseases. The Charlson comorbidity index (CCI), a validated prognostic tool, assigned weighted scores to 19 chronic diseases, based on their impact on one-year mortality [18]. Polypharmacy was defined as concurrent intake of ≥ 5 medications [19]. The PRISCUS list, tailored to elderly patients in Germany, identifies 187 potentially inappropriate medications to mitigate adverse drug reactions [14, 16]. For drug classification the globally recognized Anatomical Therapeutic Chemical (ATC) classification system was applied. The ATC offers several key advantages by providing a standardized, hierarchical structure for categorizing drugs based on their anatomical site of action, therapeutic use, pharmacological properties, and chemical characteristics, which facilitates consistent communication and comparison across different healthcare systems and countries [20–22].

Drug interaction was classified using the “MMI-AMTS-Service,” a validated system by Vidal MMI Germany GmbH, approved under Regulation (EU) 2017/745 (Medical Device Regulation) [23–25], categorizing risks into four levels: none, low, moderate and severe.

### Statistical analysis

Data management and analysis were performed using Microsoft Excel (Microsoft, Redmond, WA, USA) and SPSS Statistics v27.0 (IBM, Armonk, NY, USA). A p value of < 0.05 was considered statistically significant. No adjustments were made for multiple comparisons to prevent misinterpretation, and p-values are reported descriptively. Standard deviation (SD) summarized the variability of continuous variables around the mean.

Survival analyses employed the Kaplan-Meier method to estimate OS and disease-free survival (DFS). OS was defined as the time from diagnosis or treatment initiation to death from any cause or last follow-up. DFS was the duration from diagnosis to recurrence or death.

The Cox proportional hazards model quantified associations between predictors and survival, estimating hazard ratios (HR) and 95% confidence intervals (CI) using univariate and multivariate models. Interaction terms were incorporated, with likelihood ratio tests assessing statistical significance.

Logistic regression evaluated associations between explanatory variables and categorical outcomes, with Odds Ratios (OR) and 95% CI quantifying event likelihood. Multinomial logistic regression assessed predictors, such as drug interaction risk, on mutually exclusive categorical outcomes.

### Eligibility criteria

- **Inclusion criteria**:
  - Patients aged ≥ 18 years with histologically confirmed primary oral squamous cell carcinoma.
  - Undergoing curative surgical treatment at the Department of Oral and Maxillofacial Surgery, Charité - Universitätsmedizin Berlin, between January 2017 and December 2022.
  - Availability of complete clinical documentation, including preoperative medication history andcomorbidity profile.
  - Minimum postoperative follow-up of 6 months.
- **Exclusion criteria** **:**
  - Incomplete tumor resection (R2 or unknown margin status).
  - Presence of synchronous or metachronous secondary malignancies.
  - Distant metastases (M1 stage) at the time of diagnosis.
  - Prior radiation therapy to the head and neck region.
  - Missing data on pharmacological treatment, comorbidities, or outcome parameters.

## Results

### Patient characteristics

A total of 111 men (58.4%) and 79 women (41.6%) were included. The mean age at diagnosis was 64 ± 11.6 years, with 98 patients (51.6%) younger than 65 years and 92 (48.8%) older.

Analysis of tumor location showed that the most common site was the floor of the mouth (32.1%, *n* = 61), followed by the lower gingiva (27.4%, *n* = 52), and the tongue (25.8%, *n* = 49). Among those with metastatic involvement, 5.3% had a single metastasis, 3.7% had two, and 3.1% had three or more. All patients were initially staged as M0 according to the TNM classification system (AJCC 8th edition). The metastases reported in this study occurred during the clinical course and were categorized as part of the disease progression or recurrence. In the multivariate Cox regression model, the presence of metastases was associated with a significantly reduced OS (HR = 2.7, 95% CI:1.4–5.3). Table 1 shows the TNM classifications (AJCC 8th Edition) and UICC staging. In the present study, adjuvant radiotherapy, administered to 52% of patients, had no significant impact on DFS or OS, and while no explicit data on complications were available, the fact that all patients completed radiotherapy suggests that treatment-related adverse effects were manageable.

**Table 1:** Summary of clinical and histopathological TNM classifications (AJCC 8th Edition), UICC staging, and tumor-specific characteristics in our study cohort

Parameter	*n* (%)	Parameter	*n* (%)
cT stage		pT stage	
cT1	22 (11.6)	pT1	30 (15.8)
cT2	62 (32.6)	pT2	48 (25.3)
cT3	25 (13.2)	pT3	46 (24.2)
cT4	4 (2.1)	pT4a	64 (33.7)
cT4a	75 (39.9)	pT4b	2 (1.1)
cT4b	1 (0.5)		
cT4c	1 (0.5)		
cN stage		pN stage	
cN0	21 (11.1)	pN0	78 (41.1)
cN1	63 (33.2)	pN1	33 (17.4
cN2	14 (17.4)	pN2	1 (0.5)
cN2a	21 (11.1)	pN2a	5 (2.6)
cN2b	40 (21.1)	pN2b	19 (10.0)
cN2c	25 (13.2)	pN2c	4 (2.1)
cN3a	1 (0.5)	pN3a	1 (0.5)
cN3b	5 (2.6)	pN3b	49 (25.8)
cM stage		pM stage	
cM0	190 (100.0)	pM0	190 (100.0)
cUICC		pUICC	
I	9 (4.7)	I	19 (10.0)
II	7 (3.7)	II	20 (10.5)
III	47 (24.7)	III	45 (23.7)
IVa	121 (63.7)	IVa	58 (30.5)
IVb	4 (2.1)	IVb	48 (25.3)
IVc	1 (0.5)		
R0 resection	180 (95)	Adjuvant Radiotherapy	99 (52)
R1 resection	10 (5)	No adjuvant Radiotherapy	91 (48)
Metastases	23 (12)		
No metastases	167 (88)		

Lifestyle analysis showed that 112 patients (58.9%) were smokers, with a median tobacco consumption of 39.1 ± 19.7 pack-years (range 6–100), and 76 (40%) reported chronic alcohol abuse.

Median OS was 31.0 ± 20.9 months, with survival rates of 80.4% at 1 year. Median DFS was 30.0 ± 21.7 months, with rates of 76.6% at 1 year, 70.3% at 3 years and 58.4% at 5 years.

### Multimorbidity

**Fig. 1:**
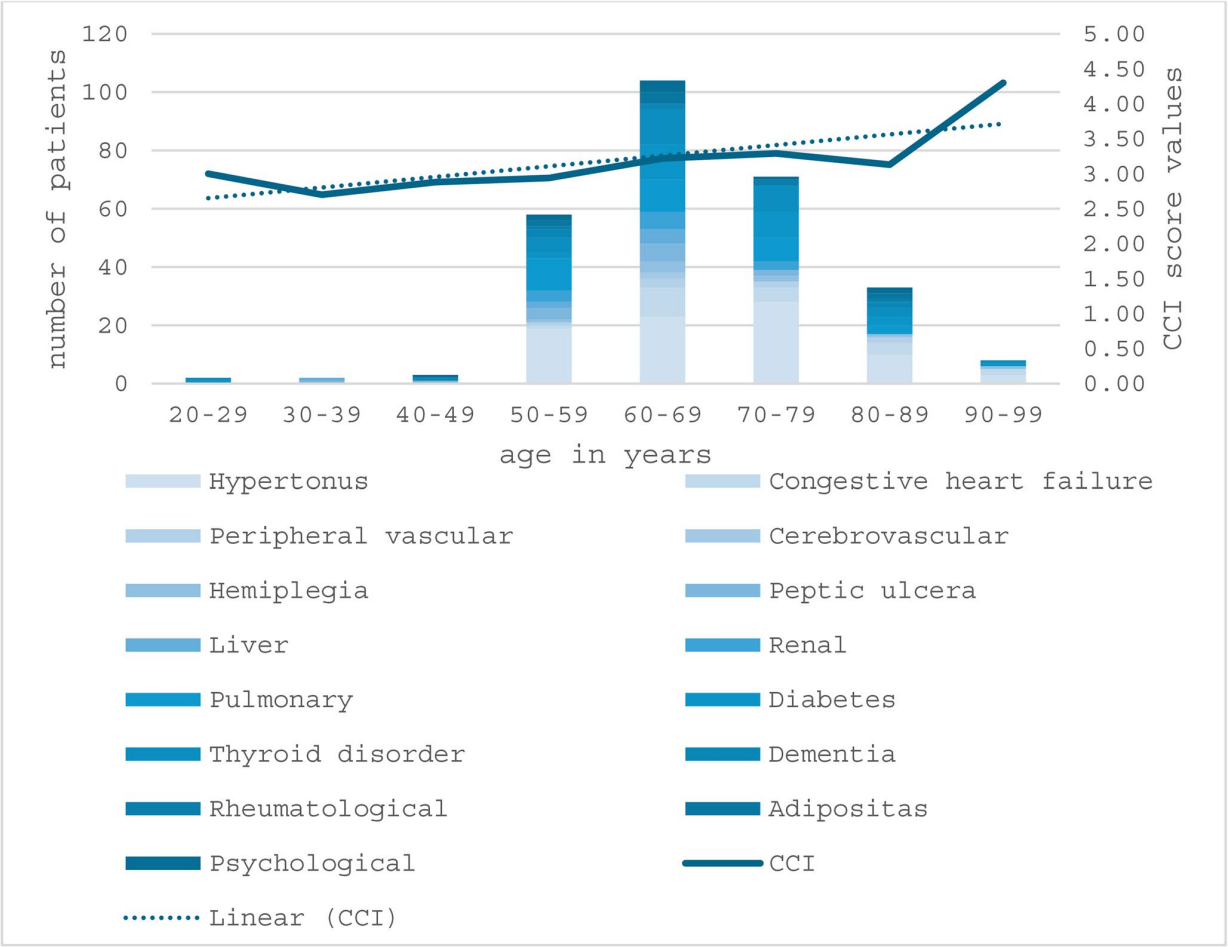
Age distribution of patients stratified by comorbidities and corresponding Charlson comorbidity index (CCI) scores. The stacked bars represent the number of patients with specific comorbidities across age groups, while the line graph illustrates the mean CCI score per age category

Multimorbidity was observed in 72 patients (37.9%). Figure 1 illustrates the comorbidity distribution by age and trends in the CCI, where higher CCI scores indicate greater comorbidity burden and mortality risk. A detailed overview of all comorbidities observed with their ICD-10 code is provided in Table 2.

**Table 2:** Prevalence of comorbidities in the study cohort, presented as absolute numbers (n) and percentages (%), reflecting the Multimorbidity burden within the population

Morbidity	ICD-10	*n* (%)	Morbidity	ICD-10	*n* (%)
Arterial hypertension	I10	84 (44.2)	Adipositas	E66	8 (4.2)
Congestive heart failure	I50	22 (11.6)	Dementia	F00-F03	8 (4.2)
Hypothyreosis	E00-07	20 (10.5)	Mild liver disease	K70-77	6 (3.2)
COPD	J44	20 (10.5)	Depression	F32-33	6 (3.2)
Peptic ulcer disease	K25, K26	14 (7.4)	Prior myocardial infarction	I25.2	5 (2.6)
Severe renal disease	N18	10 (5.2)	Hypokalemia	E87.6	4 (2)
Hemiplegia	G81	10 (5.2)	Deficiency anemia	D53.9	4 (2)
Peripheral vascular disease	I73.9	9 (4.7)	Psychosis	F20-29	3 (1.5)
Cerebrovascular disease	I60-69	2 (1)	HIV	B24	3 (1.5)
AIDS	B20-23	1 (0.5)	Severe liver disease	K72/K74.6	2 (1)

Patients with multimorbidity had a median OS of 29.5 ± 19.5 months (95% CI: 24–33) and median DFS of 26 ± 20 months (95% CI: 21.1–30.4), with 3-year OS of 52.5% and 5-year OS of 9.3%. In contrast, patients without multimorbidity had a median OS of 33 ± 21.2 months (95% CI: 29.2–36.8) and median DFS of 32 ± 22.2 months (95% CI: 28–36).

**Fig. 2:**
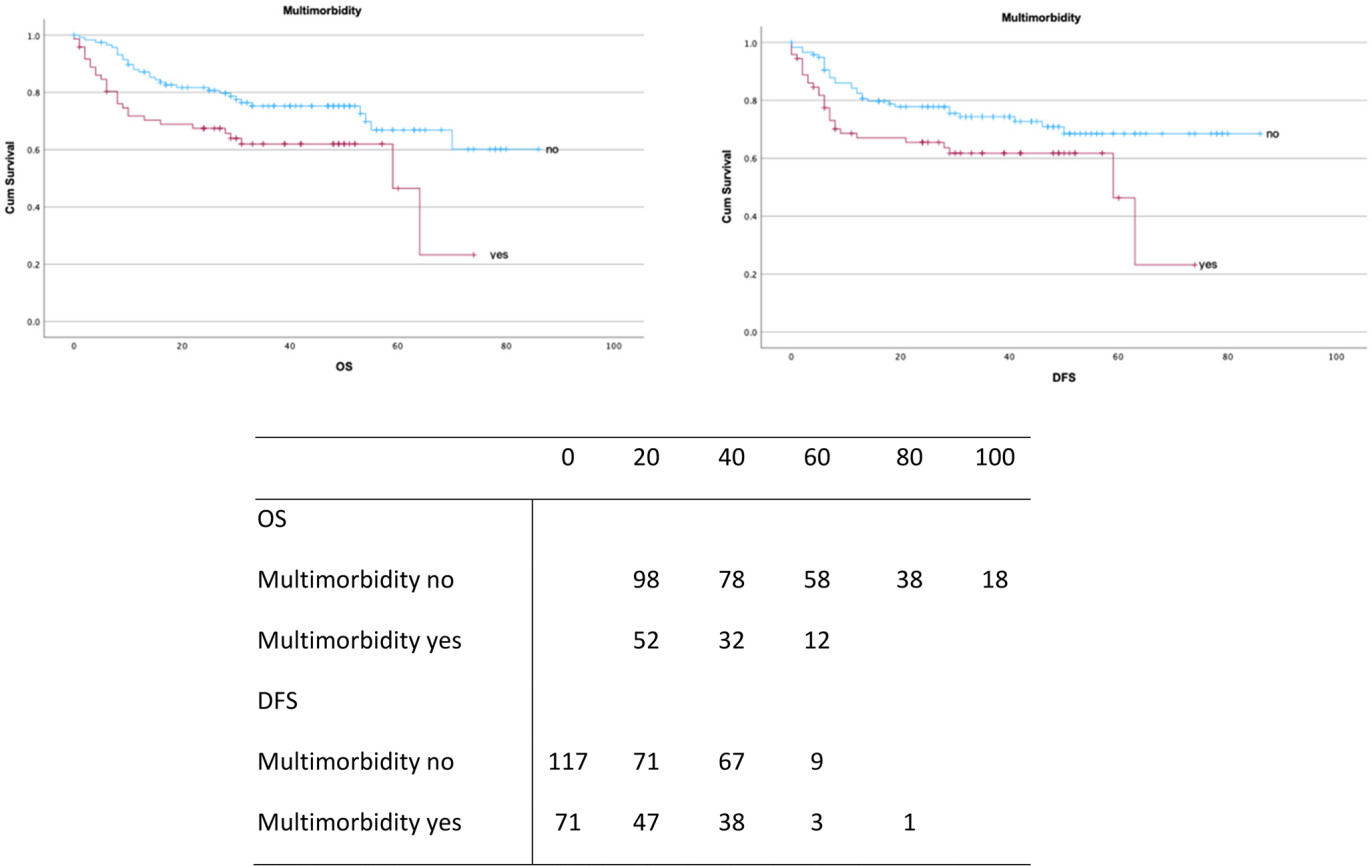
Kaplan-Meier survival curves for OS and DFS (in months) stratified by multimorbidity status. Censored observations are shown

Multimorbidity was significantly associated with reduced OS (log-rank *p* = 0.01) and DFS (log-rank *p* = 0.02), as shown in Kaplan-Meier curves (Fig. 2). Univariate Cox regression demonstrated that multimorbidity increased the risk of death (HR = 1.7, 95% CI: 1.0–3.0; Supplementary Table S1). However, in multivariate analysis (Table 3), multimorbidity did not remain an independent predictor of OS (HR = 1.5, 95% CI: 0.8–2.8) or DFS (HR = 1.4, 95% CI: 0.8–2.7).

**Table 3:** Reduced multivariate Cox regression model for OS and DFS

Variable	HR (OS)	95% CI (OS)	*p*-value (OS)	HR (RFS)	95% CI (RFS)	*p*-value (RFS)
Sex	1.895	1.069–3.359	0.029	1.243	0.796–1.941	0.339
p-Stage I	0.869	0.054–14.805	0.939	0.547	0.168–1.778	0.316
p-Stage II	4.471	0.502–34.655	0.186	1.068	0.419–2.722	0.89
p-Stage III	6.125	0.802–46.758	0.081	1.302	0.547–3.099	0.55
p-Stage IV	20.665	2.695–158.449	0.004	3.643	1.516–8.759	0.004
Age-adjusted Charlson Comorbidity Index (ACCI)	0.724	0.366–1.431	0.353	0.886	0.521–1.507	0.656
COPD	2.442	1.205–4.947	0.013	1.525	0.805–2.890	0.196
PRISCUS medication	1.542	1.006–2.365	0.047	1.194	0.847–1.684	0.311
Drug interaction risk	1.49	1.049–2.116	0.026	1.455	1.088–1.947	0.012
Polypharmacy	1.398	0.566–3.453	0.468	1.15	0.552–2.396	0.709

Among individual comorbidities, arterial hypertension (HR = 2.2, 95% CI: 1.1–3.2) and chronic obstructive pulmonary disease (COPD) (HR = 2.0, 95% CI: 1.1–4.0) were the most prevalent and significantly associated with reduced OS.

### Polypharmacy and drug interaction analysis

**Fig. 3:**
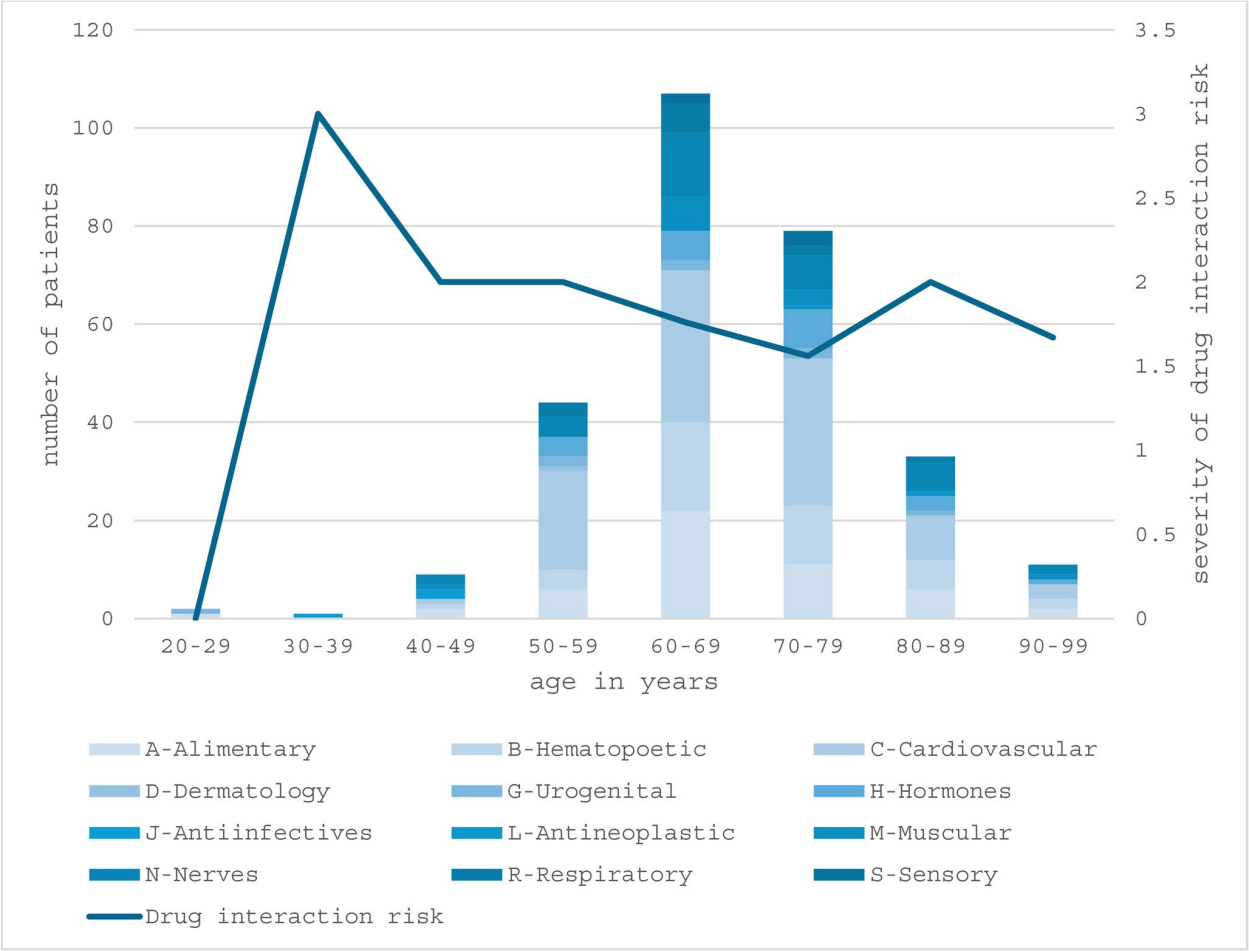
Age distribution of patients, categorized by anatomical therapeutic chemical (ATC) drug classes, and corresponding severity of drug interaction risk. The stacked bars represent the number of patients receiving medications from different ATC categories within each age group, while the line graph indicates the severity of drug interaction risk

**Fig. 4:**
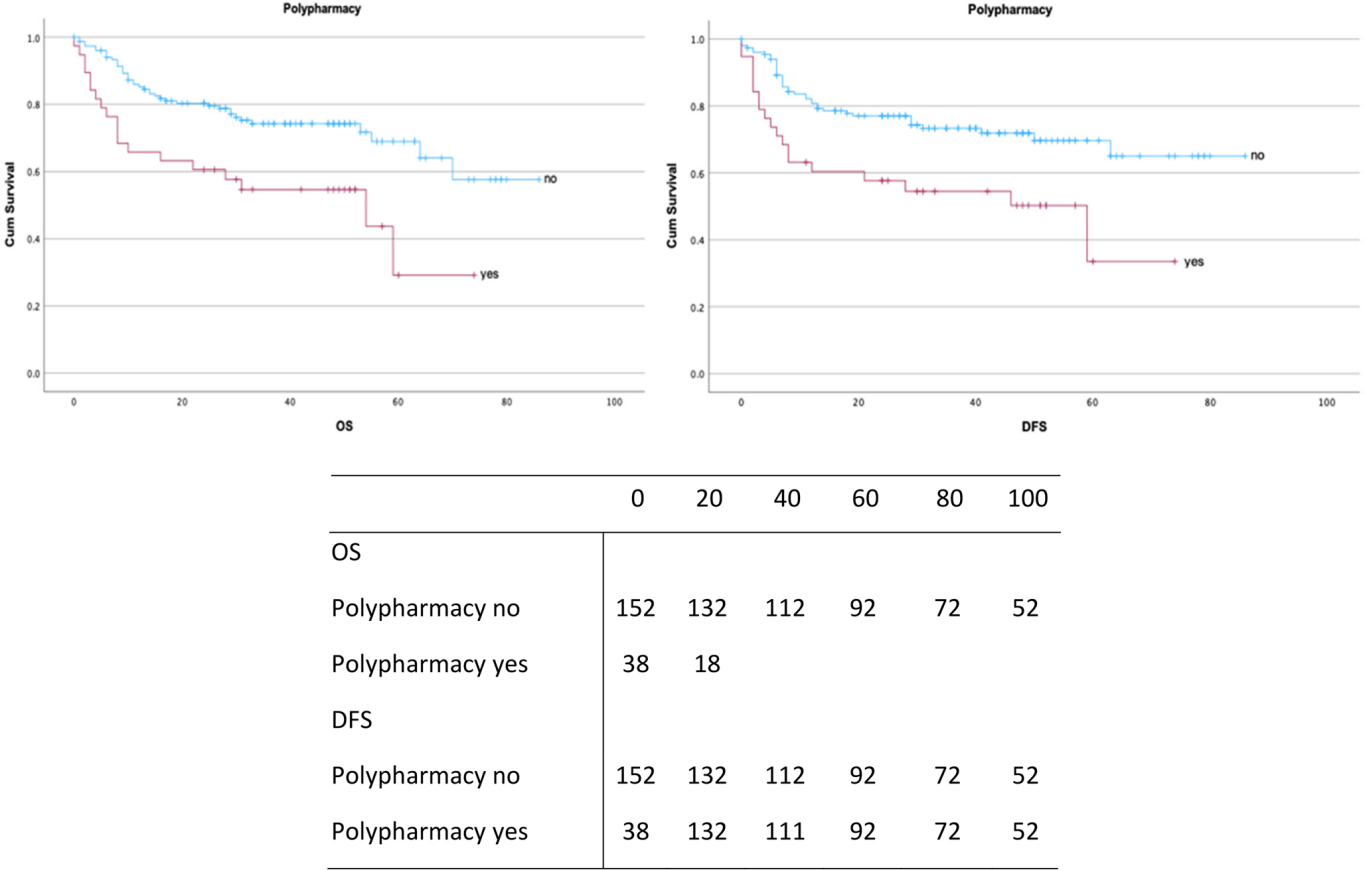
Kaplan-Meier survival curves for OS and DFS (in months) stratified by polypharmacy status. Censored observations are shown

Polypharmacy was observed in 38 patients (20%), with 37 patients (19.5%) at moderate and 8 patients (4.2%) at severe risk of drug–drug interactions. Additionally, 26 patients (13.7%) received PRISCUS-listed potentially inappropriate medications (Figs. 3 and 4**).** A comprehensive overview of prescribed medications classified according to the ATC system is presented in Table 4.

**Table 4:** Overview of medications categorized according to the anatomical therapeutic chemical (ATC) classification system at the time of initial diagnosis. Only ATC categories with recorded medication intake are listed; categories without medication intake were not observed in the study cohort

Medication	*n* (%)	Medication	*n* (%)
A02 - Drugs for Acid related disorders	20 (16)	J02 - Antimycotic drugs	1 (0.5)
A10 - Drugs used in diabetes	20 (16)	J05 - Antiviral drugs	1 (0.5)
A11 - Vitamins	6 (3)	L02 - Endocrine therapy	1 (0.5)
A12 - Mineral supplements	1 (0.5)	L04A - Immunosuppressants drugs	1 (0.5)
B01 - Antithrombotic agents	42 (22.1)	M03 - Muscle relaxants	3 (1.5)
B03 - Antianemic drugs	6 (3)	M04 - Antigout preparations	9 (4.7)
C03 - Diuretics	23 (12.1)	N01 – Anesthetics	1 (0.5)
C07 - Beta blocking agents	48 (25)	N02 – Analgesics	19 (10.5)
C08 - Calcium channel blockers	19 (10)	N03A - Antiepileptics	4 (2.1)
C09 - Agents acting on the RAAS-System	64 (33)	N04 - Antiparkinson drugs	2 (1)
D01 - Antifungals	1 (0.5)	N05 - Psychoepileptic drugs	11 (5.8)
G03 - Sex hormones	2 (1)	N06 - Psychoanaleptics	12 (6.3)
G04 - Urologicals	6 (3)	R03 - Drugs for COPD	11 (5.8)
H02 - Corticosteroids systemic	3 (1.6)	R05 - Cough drugs	1 (0.5)
H03 - Thyroid therapy	19 (10)		

Median survival was markedly reduced in polypharmacy patients (OS 29.7 months, 95% CI: 22.8–36.7; DFS 23.7 months, 95% CI: 17.0–30.4). Kaplan–Meier analyses demonstrated significantly impaired OS (*p* = 0.002) and DFS (*p* = 0.003) in this group (Fig. 4).

**Fig. 5:**
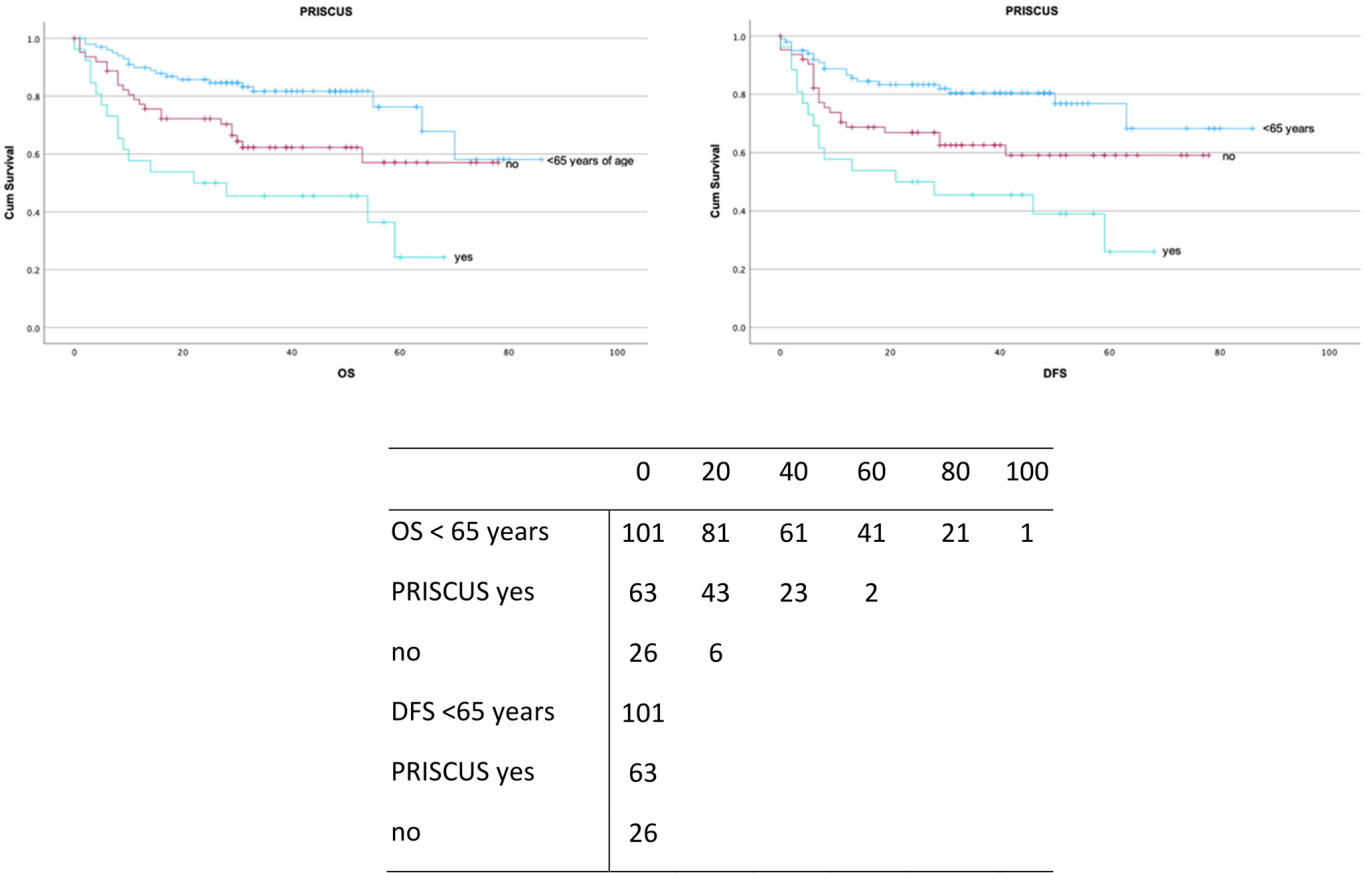
Kaplan-Meier survival curves for OS and DFS (in months), stratified by PRISCUS medication. Censored observations are indicated

Univariate Cox regression confirmed these findings, with polypharmacy associated with worse OS (HR = 2.3, 95% CI: 1.3–4.0) and DFS (HR = 2.1, 95% CI: 1.2–3.7) (Supplementary Table 2). In multivariate analysis, PRISCUS medications remained independently associated with impaired survival (HR = 1.9, 95% CI: 1.3–2.8 for OS; HR = 1.8, 95% CI: 1.2–2.7 for DFS) (Fig. 5).

**Fig. 6:**
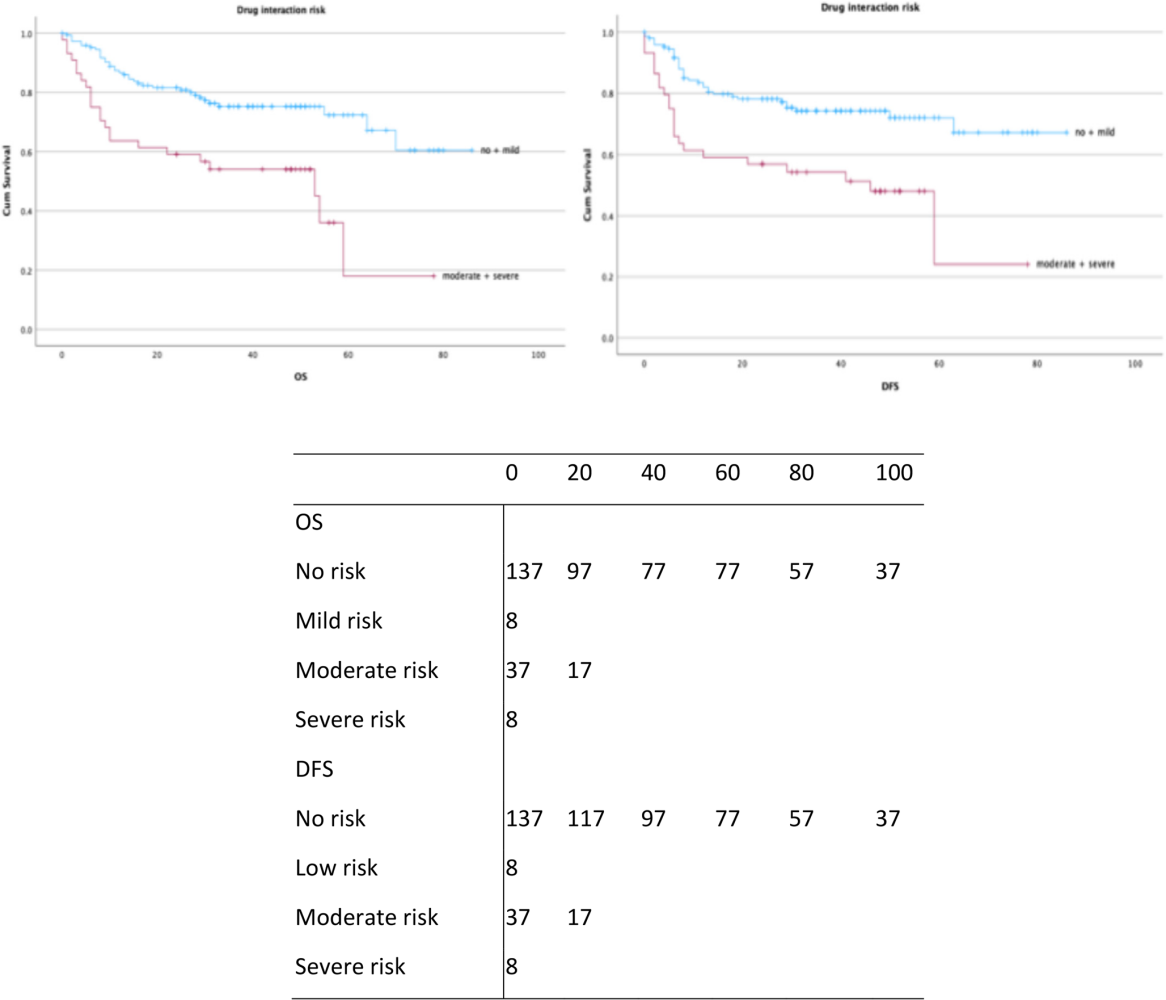
Kaplan-Meier survival curves for OS and DFS (in months), stratified by drug interaction risk. Censored observations are indicated

Drug–drug interaction risk was also significantly correlated with survival, with higher risks leading to worse OS (*p* = 0.003) and DFS (*p* = 0.006) (Fig. 6). In the multivariate model, moderate interaction risk, hormonal agents, and antineoplastic drugs were associated with poorer outcomes. Additionally, ordinal logistic regression demonstrated that polypharmacy was strongly associated with an increased risk of drug–drug interactions (*p* < 0.01).

### Treatment outcomes

A total of 88 patients (46.3%) underwent additional surgery, most frequently for wound healing complications (27.9%, *n* = 53) or for recurrence/metastatic disease (16.3%, *n* = 31). Among multimorbid patients, 40.3% required reoperations, with wound healing issues accounting for 72% of these procedures, equally distributed between tumor and donor sites.

Postoperative delirium (ICD-10: F05.8) occurred in 11 multimorbid patients (15.3%), while cardiovascular complications (27.7%, *n* = 11) were often related to antihypertensive medication causing hypotension (ICD-10: I95.2). Similar trends were seen in polypharmacy patients, where 26.3% (*n* = 10) developed cardiovascular problems, predominantly hypotension (60%).

Overall, 84 patients required readmission, most commonly for wound healing complications (21.6%, *n* = 41; ICD-10: T81.8) or recurrence-related surgery (22.1%, *n* = 42). In multimorbid patients, 29 readmissions were documented, of which 62% were due to wound healing complications. Postoperative delirium was slightly more frequent in this group (18%) compared to the overall study population (13.2%, *n* = 25). Adjuvant radiotherapy was significantly associated with higher readmission rates (*p* < 0.001).

Most patients (86.3%) were discharged home, while 13.7% required non-hospital care. Nearly all (97.9%) were admitted from home, with only 2.1% admitted from nursing facilities.

Multinomial logistic regression indicated that avoiding PRISCUS category 1 medications reduced the risk of rehospitalization by 55% and of reoperation by 52.3%. Polypharmacy patients had significantly higher odds of wound healing complications (OR 1.78 × 10⁻⁷, 95% CI: 4.08 × 10⁻⁸–7.80 × 10⁻⁷) and recurrence/metastasis (*p* < 0.001). Furthermore, a moderate drug interaction risk was strongly associated with wound healing complications (OR = 207,442,774.9, 95% CI: 18,045,602.3–2,384,653,288.0) and with reoperation for recurrence/metastasis (OR = 187,219,957.9, 95% CI: 12,327,138.1–2,843,426,622.5).

## Discussion

This retrospective study on 190 patients treated with curative intent examines the relationships between multimorbidity, polypharmacy, and drug interactions with postoperative outcomes and survival in geriatric OSCC patients. While previous research has examined these factors in general or non-cancer populations [26, 27], and other malignancies, this dataset represents a comprehensive investigation specific to OSCC, a disease with unique clinical and biological characteristics [28, 29].

The analysis of tumor location revealed that the most frequent primary site was the floor of the mouth, followed by lower gingiva and tongue. This distribution is somewhat unusual for a Western European cohort, as the tongue is typically reported as the most common primary site of oral squamous cell carcinoma [30]. This predominance of gingival tumors, although unusual for a Western European cohort, likely reflects a referral bias of our tertiary center for complex mandibular resections as well as the advanced age and multimorbidity of our patients, rather than a true epidemiological shift.

Multimorbidity, defined as ≥ 2 chronic diseases, was observed in 37.9% of the cohort and significantly reduced OS, increasing the risk of OS events by 72.5%. Similar associations have been observed across malignancies, with multimorbidity increasing mortality risk by 60% in breast [31, 32], colorectal [33], and prostate cancers [34], while the impact is weaker in lung cancer, due to its aggressiveness [35]. Consistent with prior head and neck cancer studies, cardiovascular and pulmonary diseases were significantly linked to reduced OS in OSCC [36–38]. The advanced age and comorbidity burden of this cohort, combined with the complexity of major surgery, presenting significant anesthetic challenges, necessitating meticulous perioperative management. These findings emphasize the importance of addressing cardiovascular and pulmonary conditions in geriatric cancer to improve survival outcomes and long-term functionality.

The CCI, a widely used multimorbidity tool [39], has been applied in non-small cell lung cancer [32] and colorectal cancer [40, 41]. However, in this study, the CCI was not significantly associated with OS when adjusted for OSCC-specific factors and age. This may reflect the limitations of CCI in capturing OSCC-specific risks, such as tobacco and alcohol use, which independently affect survival.

While multimorbidity was not significantly associated with either OS or DFS, its association with DFS suggests that chronic conditions may influence cancer progression through persistent inflammation, impaired immune surveillance, or changes in the tumor microenvironment. This is consistent with studies in breast, colorectal and prostate cancer [33, 42], which show that systemic comorbidities worsen DFS beyond the general impact on health. For oral cavity cancer progression, a relation to metabolic disorders or autoimmune diseases is evident. Persistent inflammation in the oral cavity leads to the recruitment and activation of immune cells, including tumor-associated macrophages (TAMs), regulatory T cells (Tregs), and neutrophils, which collectively contribute to a pro-tumorigenic microenvironment. This chronic inflammatory state promotes the release of cytokines (e.g., IL-1β, IL-6, IL-10, IL-18, TNF-α) and growth factors that facilitate tumor cell proliferation, invasion, and angiogenesis, while simultaneously impairing effective immune surveillance against malignant cells. Additionally, metabolic changes associated with aging and obesity further contribute to immunosuppression and the creation of a tumor-promoting microenvironment, characterized by increased angiogenesis and diminished antitumor immunity.

Previous studies have suggested that, in oral cavity cancer, metabolic disorders and autoimmune diseases may contribute to disease progression. Chronic inflammation in the oral cavity has been reported to recruit and activate immune cells, including tumor-associated macrophages (TAMs), regulatory T cells (Tregs), and neutrophils, which together create a pro-tumorigenic microenvironment. This inflammatory state promotes the release of cytokines (e.g., IL-1β, IL-6, IL-10, IL-18, TNF-α) and growth factors that facilitate tumor cell proliferation, invasion, and angiogenesis, while simultaneously impairing immune surveillance [43–46]. In addition, metabolic changes associated with aging and obesity have been linked to immunosuppression and the development of a tumor-promoting microenvironment, characterized by increased angiogenesis and reduced antitumor immunity [46]. However, our study does not provide mechanistic data on these processes, and such explanations should be regarded as hypotheses derived from prior literature rather than conclusions from our dataset.

Nevertheless, these findings challenge the conventional view of multimorbidity as affecting non-cancer mortality and highlight its role in cancer recurrence and metastasis. Chronic diseases can exacerbate cancer progression or reduce treatment efficacy, as seen in cardiovascular and metabolic diseases, which promote inflammation and oxidative stress and accelerate tumor growth [39].

A comprehensive multimorbidity framework is required. Studies in geriatric oncology show that frailty indices, such as the Clinical Frailty Scale [40], improve survival prediction in older cancer patients [41]. This is consistent with efforts to move from disease-centered to patient-centered cancer care.

Polypharmacy, observed in 20% of the cohort and was associated with reduced OS and DFS, consistent with literature [42] linking polypharmacy to ADEs, non-adherence and reduced resilience in geriatric patients. HRs indicate a higher likelihood of adverse outcomes in this group reinforcing its role as a modifiable risk factor in cancer management.

The analysis demonstrated strong associations between polypharmacy and specific postoperative complications, including wound healing disorders and recurrence/metastasis. These associations are likely to arise from the pharmacological effects of commonly prescribed medications within polypharmacy regimens. For instance, corticosteroids and anticoagulants, frequently used in this population, are known to delay angiogenesis [47–49] and collagen synthesis [50], while immunosuppressants exacerbate infection risk and systemic inflammation [51, 52]. These effects are of particular concern in OSCC patients, where extensive surgical interventions impose significant physiological demand and necessitate efficient wound healing and immune competence. Furthermore, ordinary logistic regression identified a relationship between polypharmacy and drug interaction risk, suggesting a potential indirect impact on treatment outcomes. This study had sufficient power (83.1%) to detect moderate effects (HR >1.5) in Cox regression, supporting key findings. However, wide confidence intervals for multimorbidity (HR = 1.5, 95%CI: 0.8–2.8) and polypharmacy (HR = 2.3, 95%CI: 1.3–4.0.3.0) suggest larger samples may improve precision and detect smaller associations. Future studies with larger cohorts could enhance these estimates and assess subgroup differences.

The findings reinforce the need for systematic preoperative medication reviews and targeted deprescribing interventions. Structured tools such as the STOPP/START criteria [53] and the Beers Criteria, established in 2003 [54, 55] can assist clinicians in optimizing pharmacological regimens, ensuring that only essential medications are maintained. The necessity for future research is highlighted, with studies requiring the implementation of longitudinal designs and randomized controlled trials to evaluate the impact of deprescribing interventions on survival, quality of life, and treatment efficacy in the field of geriatric oncology.

The present study has identified drug interaction risks as a relevant factor in postoperative outcomes, with moderate interaction risks showing a strong association with wound healing disorders. These findings are consistent with those reported previously, who emphasized the deleterious effects of common drug combinations in older adults, such as anticoagulants and NSAIDs, which compromised hemostasis and delay tissue repair. It is well established, that drug interactions disrupt physiological recovery pathways, including clot formation, inflammation resolution, and tissue remodeling, thereby exacerbating postoperative complications. Hormonal and antineoplastic medications have also been demonstrated to have a substantial impact on OS, likely reflecting their systemic effects, such as myelosuppression, impaired immune function, and reduced wound healing capacity. These findings align with previous research, which has indicated that oncological therapies can influence survival outcomes by increasing susceptibility to infection, delaying recovery, and exacerbating comorbid conditions.

The utilization of PRISCUS-listed medications, deemed potentially inappropriate for older adults, was observed in 13.7% of the study population and was significantly associated with poorer survival outcomes. Specifically, PRISCUS medications were associated with worse OS and DFS. These findings are consistent with existing literature, which has demonstrated that potentially inappropriate medications (PIMs) increase the risk of Adverse Drug Events (ADEs), polypharmacy, and mortality in geriatric patients [56–59]. Studies have shown that PIMs can impair organ function, exacerbate preexisting conditions, and disrupt the pharmacokinetics of other medications, further complicating the clinical management of older patients. The correlation between PRISCUS-listed medications and diminished survival underscores the pivotal necessity for adherence to evidence-based geriatric prescribing practices. Prior studies have emphasized the merits of tools such as STOPP/START criteria in reducing inappropriate prescribing.

These frameworks emphasize the importance of deprescribing, medication reconciliation, and the substitution of high-risk medications with safer alternative to mitigate the risk of AEDs and improve patient outcomes.

The study also identified a potential protective effect of avoiding PRISCUS medications in older adults. Patients over the age of 65 who did not receive medications listed on the PRISCUS-listed medications exhibited a 55% reduction in the likelihood of rehospitalization and a 52.3% reduction in the risk of reoperation. These findings corroborate earlier reports, which highlighted that avoiding PIMs can reduce healthcare utilization and improve survival outcomes in older patients. The results emphasize that avoiding PIMs may also reduce healthcare-associated complications, such as infections and readmissions, while enhancing recovery and maintaining functional independence.

The proactive implementation of these tools upfront for treatment guidance appears to be a rational and beneficial strategy. However, it is important to acknowledge, that certain high-risk patients may still experience suboptimal treatment responses. In cases where medication adjustments cannot fully mitigate risks, a multidisciplinary approach remains essential to balancing oncological efficacy with patient safety.

The high rates of reoperations (46.3%) and rehospitalizations (46.3%) in patients older than 65 years old, highlight the significant challenges of managing geriatric OSCC patients. Wound healing complications (27.9% of reoperations and 21.6% of rehospitalizations) and reoccurrence-related procedures (16.3% and 22.1%, respectively) were the most frequent indications. These findings are consistent with literature that highlights the physiological impairments associated with aging, which adversely affect surgical outcomes [60–63].

Patients, who were not prescribed medications within the PRISCUS categories, exhibited lower rates of rehospitalization and reoperation. This finding highlights the importance of a nuanced approach to prescribing, balancing the potential harms of potentially inappropriate medications with their ability to address specific perioperative needs.

The utilization of structured prescribing tools can facilitate optimization of medication regimens.

The elevated rates of reoperations and rehospitalizations in geriatric OSCC patients underscore the intricate interplay of aging, comorbidities, and cancer-related factors in determining postoperative outcomes.

This study underscores the importance of integrating pharmacological and geriatric assessments into routine perioperative care for older adults with OSCC. Key recommendations include:

1. Comprehensive medication optimization: Preoperative medication reviews should systematically address polypharmacy and potential drug interactions, aiming to subsequently initiate deprescribing interventions.
2. Frailty and functional assessments: Incorporating frailty measures (e.g., Clinical Frailty Scale [64], modified Frailty index with 5 items (mFI-5) [65]) into risk stratification could enhance predictions of surgical outcomes beyond multimorbidity alone.
3. Multidisciplinary care approach: Close collaboration among surgeons, geriatricians, and pharmacists is crucial to tailoring pharmacological and perioperative strategies ensuring that individual patient vulnerabilities are addressed comprehensively.

### Limitations and future research

The retrospective design allowed for a comprehensive dataset but limits causal inferences. Reliance on existing clinical records introduces potential documentation bias, affecting data accuracy and completeness. Missing documentation on specific causes of death precluded time-conditional probability analysis, preventing identification of when non-cancer causes surpass index cancer deaths. Additionally, the study’s single-center setting limits generalizability. Further prognostic factors such as the nutritional status (e.g., BMI) were not assessed, despite their potential impact on OSCC prognosis. Likewise, treatment modifications in polypharmacy patients were not analyzed, leaving uncertainty about whether clinical decisions were adapted based on medication burden. The focus on selected clinical parameters excluded molecular markers, which could have provided further prognostic insights. The logistic regression results, particularly those with wide confidence intervals, require validation in larger, more diverse cohorts. In the present study, the impact of polypharmacy, multimorbidity, and drug interactions on postoperative outcomes was not explicitly analyzed in the 52% of OSCC patients who received adjuvant radiotherapy. The impact of surgical complications on treatment completion and therapy-related toxicity (e.g., mucositis, dysphagia, skin reactions, hematologic toxicity) could not be fully assessed due to limited documentation on adjuvant therapy adherence and side effects. While these factors are likely to influence postoperative recovery and overall treatment tolerance in this subgroup, our primary focus was on the general postoperative risks associated with multimorbidity, polypharmacy and drug-interactions. A separate, dedicated analysis would be necessary to assess how these variables specifically interact with adjuvant radiotherapy, its associated complications or could contribute to the refinement of treatment strategies and ultimately improve patient outcomes. These gaps highlight the need for future prospective studies with comprehensive datasets. Prospective studies and randomized controlled trials on deprescribing interventions and multidisciplinary care models in geriatric oncology could generate high-quality evidence, ultimately refining clinical practice guidelines for this vulnerable population.

## Conclusion

This study underscores the pivotal function of multimorbidity, polypharmacy, and drug interactions in determining postoperative outcomes and survival in geriatric OSCC patients. By con-textualizing these findings within the context of current literature, the necessity for tailored perioperative strategies that integrate pharmacological reviews, detailed geriatric assessments, and coordinated multidisciplinary care is emphasized. These approaches are essential to improve both survival and quality of life for older adults undergoing curative treatment for OSCC.

## Supplementary Information

Below is the link to the electronic supplementary material.

Supplementary Material 1

## Data Availability

No datasets were generated or analysed during the current study.
