# Supplementary material for: Retrospective analysis of multimorbidity, polypharmacy, and drug interactions on postoperative outcomes in oral squamous cell carcinoma patients
Source: Clin Oral Investig. 2025 Oct 20;29(11):517. doi: 10.1007/s00784-025-06588-8 (PMC12537594; doi:10.1007/s00784-025-06588-8)
Supplement: Supplementary file 1 — Supplementary Material 1 [file 784_2025_6588_MOESM1_ESM.docx]

Supplementary materials

Supplementary Table 1: Univariate Cox regression analysis for OS and RFS, presenting HR and 95% CI for multimorbidity and individual comorbidities.

|  |  | **OS** |  |  | **DFS** |  |
| --- | --- | --- | --- | --- | --- | --- |
|  | HR | 95% CI | P-value | HR | 95% CI | P-value |
| Multimorbidity | 1.725 | 1.001-2.973 | 0.014 | 1.473 | 0.838-2.589 | 0.178 |
| CCI | 1.171 | 1.016-1.350 | 0.029 | 1.177 | 1.019-1.360 | 0.027 |
| AACCI | 1.184 | 1.059-1.325 | 0.003 | 1.172 | 1.045-1.316 | 0.007 |
| CCI excl. Primary OSCC | 1.173 | 1.018-1.351 | 0.028 | 1.179 | 1.021-1.361 | 0.025 |
| Art. Hypertension | 1.763 | 1.054-2.949 | 0.031 | 1.499 | 0.881-2.552 | 0.135 |
| Chronic pulmonary disease | 2.156 | 1.141-4.075 | 0.018 | 2.103 | 1.108-3.991 | 0.023 |
| DM w/o complications | 1.958 | 1.057-3.627 | 0.033 | 1.875 | 0.995-3.465 | 0.052 |
| Adipositas | 3.48 | 1.382-8.764 | 0.024 | 3.37 | 1.334-8.51 | 0.01 |

Supplementary Table 2: Univariate Cox regression analysis for OS and DFS, presenting HR and 95%CI for polypharmacy. Drug classification is based on the Anatomical Therapeutic Chemical (ATC) system.

|  |  | **OS** |  |  | **RFS** |  |
| --- | --- | --- | --- | --- | --- | --- |
|  | HR | CI | P-value | HR | CI | P-value |
| Polypharmacy | 2.307 | 1.334-3.990 | 0.003 | 2.112 | 1.210-3.686 | 0.008 |
| PRISCUS medication | 2.042 | 1.468-2.839 | <0.001 | 1.862 | 1.324-2.618 | <0.001 |
| Drug interaction risk - low | 0.814 | 0.198-3.338 | 0.775 | 0.683 | 0.166-2-809 | 0.598 |
| Moderate | 1.980 | 1.125-3.484 | 0.018 | 1.806 | 1.008-3.235 | 0.047 |
| Severe | 1.843 | 0.666-5.1 | 0.239 | 2.579 | 0.922-7.210 | 0.071 |
| A - Alimentary | 1.429 | 0.82-2.488 | 0.208 | 1.344 | 0.764-2.363 | 0.305 |
| B - Blood | 0.966 | 0.372-2.509 | 0.943 | 0.864 | 0.324-2.307 | 0.771 |
| C - Cardiovascular | 0.823 | 0.108-6.249 | 0.851 | 0.753 | 0.099-5.733 | 0.784 |
| G - Urogenital | 0.796 | 0.105-6.033 | 0.825 | 0.668 | 0.088-5.084 | 0.697 |
| H - Hormones | 2.847 | 1.054-7.692 | 0.039 | 3.152 | 1.157-8.589 | 0.025 |
| L - Antineoplastic, | 11.571 | 1.203-111.273 | 0.034 | 8.177 | 0.913-73.204 | 0.060 |
| M - Musculoskeletal | 0.905 | 0.297-2.759 | 0.860 | 0.920 | 0.299-2.828 | 0.884 |
| N – Nervous system | 1.723 | 0.665-4.466 | 0.263 | 1.541 | 0.568-4.180 | 0.396 |
| R - Respiratory | 1.971 | 0.414-9.392 | 0.394 | 2.055 | 0.435-9.706 | 0.366 |
